# Examining enablers of vaccine hesitancy toward routine childhood and adolescent vaccination in Malawi

**DOI:** 10.1186/s41256-022-00261-3

**Published:** 2022-08-18

**Authors:** Gbadebo Collins Adeyanju, Cornelia Betsch, Abdu A. Adamu, Khadijah Sanusi Gumbi, Michael G. Head, Aristide Aplogan, Haoua Tall, Tene-Alima Essoh

**Affiliations:** 1 Media and Communication Science, University of Erfurt, Erfurt, Germany; 2 Center for Empirical Research in Economics and Behavioural Science (CEREB), University of Erfurt, Erfurt, Germany; 3 Bernard Nocht Institute of Tropical Medicine (BNITM), Hamburg, Germany; 4 Cochrane South Africa, South African Medical Research Council, Cape Town, South Africa; 5 Division of Epidemiology and Biostatistics, Department of Global Health, Faculty of Medicine and Health Sciences, Stellenbosch University, Cape Town, South Africa; 6 Ahmadu Bello University, Zaria, Kaduna Nigeria; 7 Clinical Informatics Research Unit, Faculty of Medicine, University of Southampton, Southampton, UK; 8 Agence de Médecine Préventive, Regional Directorate for Africa, Abidjan, Côte d’Ivoire

**Keywords:** HPV, Malawi, Routine immunisation, Vaccination, Vaccine hesitancy, Vaccine uptake, Childhood, Adolescent

## Abstract

**Background:**

The contribution of vaccination to global public health and community wellbeing has been described as one of the greatest success stories of modern medicine. However, 13.5 million children still miss at least one of their routine vaccinations, and this contributes to about 1.5 million deaths from vaccine-preventable diseases. One of the contributing factors has been associated with vaccine hesitancy. Vaccine hesitancy is the delay or refusal of vaccines despite their availability. The study explored factors from multiple perspectives that influence hesitancy among caregivers of children and adolescent girls eligible for childhood routine immunisation and the Human Papillomavirus vaccine in Malawi.

**Methods:**

The methodology used was qualitative such as key informant interviews and focus-group discussion. Information was obtained from caregivers, community and religious leaders, leaders of civil society groups, teachers in schools where Human Papillomavirus vaccine were piloted, healthcare workers, national and district-level officials of the expanded program on immunisation. There were 25 key informant interviews and two focus-group discussions, with 13 participants. The study was conducted between April to May 2020. The Interviews and discussions were audio-recorded, transcribed, and analysed using a thematic content approach.

**Results:**

Most vaccine-hesitancy drivers for routine immunisation were also relevant for the HPV vaccine. The drivers included inadequate awareness of the vaccination schedule, rumours and conspiracy theories exacerbated by religious beliefs, low literacy levels of caregivers, distance and transport to the vaccination clinic, gender role and a disconnect between community healthcare workers and community leaders.

**Conclusions:**

The study demonstrated that a network of factors determines vaccine hesitancy for childhood Routine Immunisation and Human Papillomavirus, and some of them are interrelated with one another. This has implications both for current levels of vaccine acceptance and the introduction of any new vaccine, such as those against Malaria, HIV/AIDS, HPV or COVID-19 (coronavirus disease 2019). Therefore, strategies developed to address vaccine hesitancy must be multi-component and wide-ranging.

## Introduction

The contribution of vaccination to global public health and community wellbeing is one of the greatest success stories of modern medicine [1]. However, 13.5 million children missed at least one of their routine vaccinations in 2018, and this contributed to about 1.5 million deaths from vaccine-preventable diseases [2]. In the Sub-Saharan Africa (SSA) region [3], basic vaccination coverage (DTP3) is 76%, which is the lowest among World Health Organisation (WHO) member regions and 10% lower than the global average of 86% [4].

In Malawi, the overall vaccination uptake has typically been higher than much of SSA but there are still gaps in coverage. For example, the uptake of DTP3 and first dose of measles vaccine in 2018 were both 92%; however, this dropped to 75% for the second dose [5]. For newly introduced vaccinations, there was a 91% and 89% uptake for rotavirus and pneumococcal vaccine respectively. The Human Papillomavirus (HPV) vaccination showed a drop from the first to the second dose, for example, in the districts of Rumphi (98–88%) and Zomba (89–76%) [6, 7]. Vaccine hesitancy is discussed as one reason for difficulties in maximizing vaccination uptake [8].

Vaccine hesitancy is defined as the delay or refusal of vaccines despite the availability of vaccination services [8]. It has been labelled as one of the ten threats to global public health in 2019 [9], and is influenced by many factors, including safety concerns, rumours and conspiracy theories, fears of adverse events [10–12]. Much of the evidence around hesitancy has been focused on high-income settings, and little is known about the extent of, and reasons for hesitancy in lower income settings [12, 13]. The drivers can be different when introducing new vaccines such as against HPV and or COVID-19 [14, 15]. Exploring these potential differences in drivers is crucial, as knowledge about these factors will improve the chances of a successful implementation of new vaccines.

Since the introduction of a vaccine against HPV in 2013 through the pilot phase in Malawi and consequently into the Expanded Program on Immunisation (EPI) routine program in January 2019, there is emerging evidence that hesitancy may negatively affect uptake [10, 16]. HPV vaccine introduction aimed to address the increasing risks of cervical cancer incidences and associated mortality in Malawi [7]. Cervical cancer constitutes the fourth most-common form of cancer globally among women [17, 18]. Out of the 185 countries with the prevalence of cervical cancer in 2018, more than 90% were in the African region [19, 20]. Malawi has the highest incidence of cervical cancer in the world with a 50% mortality rate among women aged 15–44 years, hence, the importance of introducing HPV vaccine into the national recommended vaccination [18, 21, 22].

Studies in 13 SSA countries (not including Malawi) show that decreased access to healthcare facilities is a barrier to HPV vaccination uptake, perceived risks of receiving the vaccine, safety and effectiveness concerns, low levels of knowledge and awareness of HPV vaccine are factors contributing to low demand of vaccination [23–25]. Preliminary pilot studies have suggested that similar drivers could also be relevant in Malawi [26, 27].

The Government of Malawi Health Sector Strategic Plan II (2017–2022) has set a goal “of ensuring that the people of Malawi attain the highest possible level of health and quality of life” [28]. This will be achieved by ensuring universal coverage of basic health care, which is the obligation of the government guaranteed under the republican constitution. One of the key objectives of this plan is to reduce the burden of communicable diseases by rolling out nationwide immunisation programs.

The organization that has been leading this nationwide immunisation programs is the EPI. The EPI was officially created in Malawi in 1979, following the global launch in 1974 to ensure that children across all countries benefit from life-saving measures provided by vaccines [1]. Today, through the efforts of EPI, views of vaccines as one of the safest, cost-effective, and most successful public health interventions for vaccine preventable diseases (VPDs) has been recognised [28]. In addition, through synergy with other important stakeholders e.g., community and religious leaders, to control VPDs and achieve better health for children in all populations everywhere, the EPI goal of universal access to all relevant vaccines for children and other at-risk groups have made significant progress. However, as highlighted above, this progress has been stalled due to factors linked to vaccine hesitancy [23–27].

In Malawi, there is a paucity of evidence to support the EPI and national government’s efforts to achieve the Health Sector Strategic Plan II using evidence-based strategies. Therefore, the goal of this study was to understand factors that enable vaccine hesitancy through the lenses of vaccination stakeholders and community members in Malawi. The study considered it vital to collect opinions from both demand-side (community members including caregivers) and supply-side (healthcare providers such as the EPI and other actors), to enable comprehensive understanding of the phenomenon. The study focus is on recommended routine immunisation and HPV vaccine.

## Methods

### Study design

The study was qualitative and used Key Informant Interviews (KII) and multi-stakeholders Focus Group Discussions (FGD) techniques. The study considered exploring evidence from both key informants and the assembly of community members, not only to corroborate evidence but also to explore the dynamics of the relationship between vaccine demand and supply. The FGD provided an in-depth understanding of contextual and social issues, while KII generated knowledge insights into the phenomena under study [29].

### Study settings

**Fig. 1:**
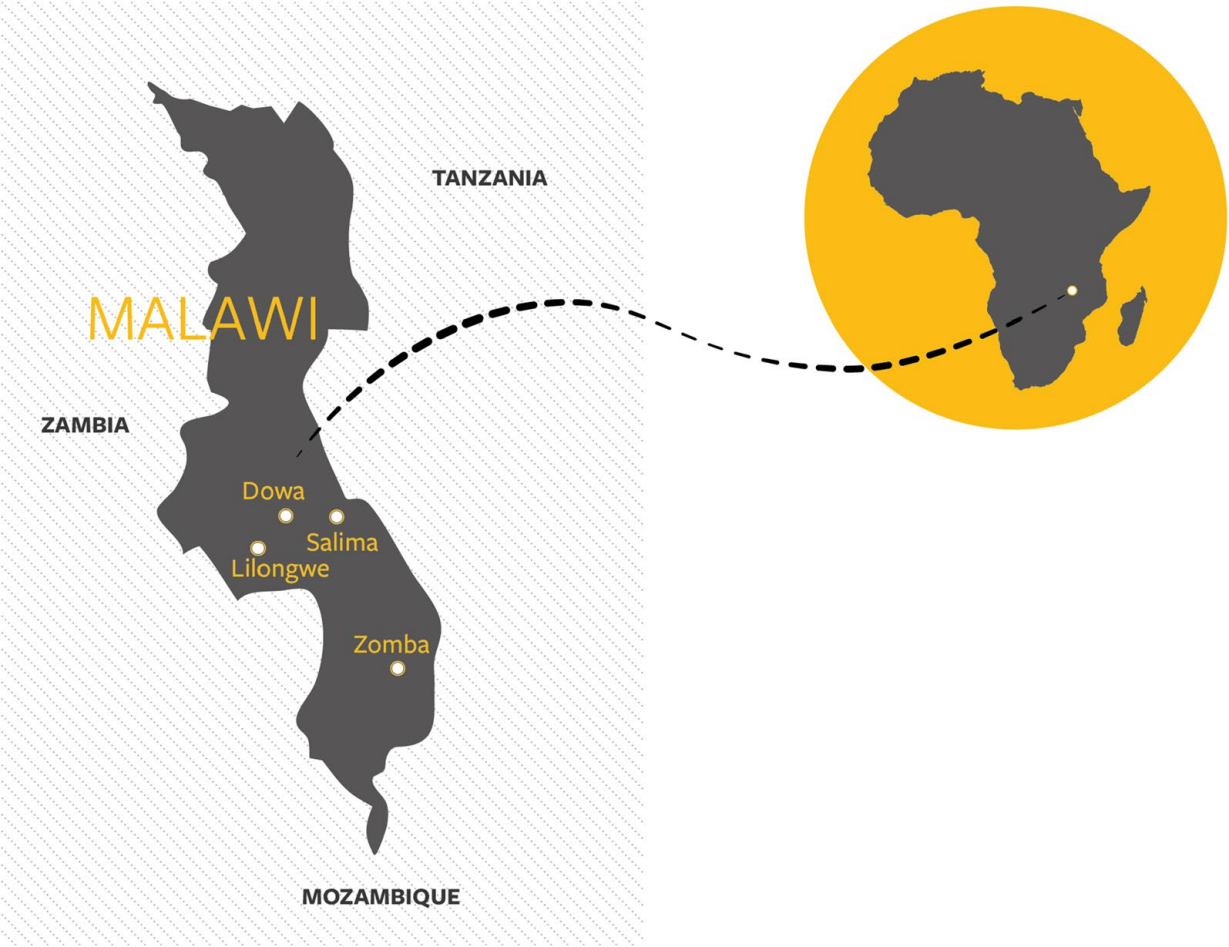
Map of study locations in Malawi

The study was conducted in the Salima, Lilongwe, Dowa, and Zomba districts of Malawi (Fig. 1). The districts were selected based on the following criteria: one district with high vaccine coverage (Lilongwe), one with low vaccine coverage (Dowa), and one each in an urban (Zomba) and rural (Salima) districts where the HPV vaccine had been implemented. The two latter districts were selected not based on coverage but on the rural–urban difference.

### Themes explored/interview outlines development

The study implementation instruments were interview guides. The guide comprised the following themes and topics, based on validated measures in the same setting [26, 27, 30]: knowledge of immunisation (“What do you know or think about immunisation?”); knowledge of HPV/cervical cancer (“Do you know or have you heard about cervical cancer and/or the HPV vaccine?”); attitude toward childhood RI and the HPV vaccine (“Do you think childhood RI is an important topic for you and/or your community?…How?” and “What do you think about the HPV vaccine and the target group?…Why do you think that way?”); barriers against childhood RI (“Do you know about vaccine hesitancy and what are the reasons for low childhood immunisation demand?…Please describe why”); barriers against HPV vaccine acceptance (“Do you know about hesitation against HPV vaccine?…If so, what are they, among whom are they, and why?”). These measures have been tested across different settings in SSA and has generated high reliability, especially in Malawi.

### Data collection

The study participants were purposively selected as shown in Table 1 below. The purpose was to target key actors with valuable insights and information, from both the demand (community) and supply (healthcare providers) sides.

**Table 1:** Composition of study participants

Method	Central level	Low/high coverage districts	Urban/rural HPV demonstration districts
KII	National EPI manager	District EPI officers	District EPI officers
	EPI social mobilisation	one community leader (CL) each	One community leader (CL) each
	One member of national immunization technical advisory group (NITAG)	one community HCW each	One community HCW each
	United Nations International Children's Emergency Fund (UNICEF) representative	EPI logistician	One representative each of religious leaders/groups (RL) or civil society organisations (CSO)
	WHO representative	One representative each of religious leaders/groups (RL) or civil society organisations (CSO)	One teacher each from a school where an hpv demonstration project was conducted
FGD			One community leader (CL) each
			One community HCW each
			One representative each of religious leaders/groups (RL)
			One representative each of civil society organisations (CSO)
			One teacher each from a school where an HPV demonstration project was conducted
			One caregiver each whose daughter was eligible for HPV vaccination (1)
			One caregiver each whose child was eligible for Routine Immunisation (2)

A total sample size of 25 key informants were interviewed, while the two FGD comprised six discussants in Zomba (urban district) and seven in Salima (rural district). For the two focus-group discussions, participants were purposively identified and selected from communities where EPI has implemented programs. Participants at the Salima FGD comprised of three females and four males, while participants at the Zomba FGD comprised of three males and three females. Each of the KII lasted 30 min on average, while each of the FGD sessions were conducted for an average time of 90 min. The interviews and discussions were voice-recorded and transcribed verbatim.

### Data analysis

The data were analysed using thematic deductive content analysis, due to its ability to test earlier assumptions in different situations or compare categories [31]. Main themes and sub-themes were identified after analysing each individual transcripts and then categorise them based on the above themes explored. Transcribed data were coded as follows: the central level was coded C001 and C002, while districts were coded as KII001 and KII002. The FGDs were coded as FGD001and FGD002. The analysis resulted in the development of a thematic index based on the themes explored, such as knowledge, attitude/perception toward vaccination, and general barriers against vaccine acceptance.

The WHO Strategic Advisory Working Group (SAGE) vaccine hesitancy model/matrix [8] was used to organise factors driving vaccine hesitancy in Malawi for both childhood RI and HPV vaccines. The model identified three dimensions for organising vaccine-hesitancy determinants: vaccine/vaccination-specific issues, individual and group influences, and contextual influences.

Vaccine/vaccination-specific issues are factors surrounding concerns about vaccines or vaccination; individual and group influences refer to individual perceptions or individuals’ social environments; and contextual influences aim at understanding vaccine hesitancy arising due to historic, socio-cultural, environmental, health system/institutional, economic, or political factors [8].

In this study, trends of factors that the participants felt most strongly about were identified. These factors were categorised under knowledge of RI, perception/attitude toward vaccinations, vaccine importance, HPV vaccine/cervical cancer knowledge and attitude, and drivers of vaccine hesitancy for childhood RI and HPV vaccine. These are important features for understanding vaccination behaviour and demand among caregivers [15, 30, 32, 33].

## Results

**Fig. 2:**
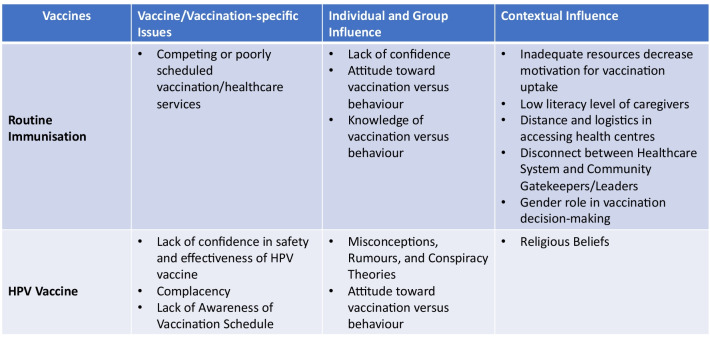
Results summary based on WHO SAGE vaccine hesitancy determinant model. *HPV* Human papillomavirus

The Fig. 2 below displays summary of study results using the vaccine hesitancy determinant model of the WHO SAGE [8].

### Factors driving vaccine hesitancy toward routine immunisation

*Competing or poorly scheduled healthcare services:* Participants reported that based on their experiences, “outreach clinics are opened most of the time around 9:00am. Sometimes outreach clinics are missed by caregivers due to other services scheduled at the same time, such as family planning and ante-natal care services” (HCW 2, Salima District). Outreach clinics are usually makeshift medical services decentralised to reach disadvantaged communities or hard-to-reach terrains to provide essential healthcare services, especially immunisation. Participants alluded to missed appointments resulting in incomplete immunisation when all the immunisation services were not centralized in one spot or operated on different schedules.

*Lack of confidence:* The study participants acknowledged the presence of RI and other types of immunisation services that are being provided in Malawi. However, most participants reported that despite at least five healthcare workers providing services at the facilities, which was adequate for the setting, the number of adolescent girls and under-fives receiving vaccination remained low in some districts. An FGD participant alluded to confidence issues: “Many people do not trust immunisation because of stories they hear” (EPI Manager, Zomba District).

*Attitude toward vaccination versus behaviour:* Study participants acknowledged that vaccination is vital to caregivers for protecting their children (those under-five) against vaccine-preventable diseases and agreed that vaccination is a vital topic within their household. However, immunisation was not considered a top priority: “immunisation is very important but there are other equally important things to the family” (Caregiver 2—Salima District).

*Inadequate resources decrease motivation for vaccination uptake:* Most participants reported lack of resources and medical equipment at village clinics. This necessitates caregivers and HCWs to sacrifice their personal items for immunisation activities, hence reducing their motivation and willingness to want to continue immunisation. “We use our own resources (e.g., transportation, sanitary items, furniture, etc.) whenever we want to do vaccination activities at the village” (HCW 2, Lilongwe).

*Low literacy level of caregivers:* The participants also revealed that caregivers’ literacy levels are very low in the communities. This makes effective health promotion of issues through written materials very difficult: “Sometimes some people distribute pamphlets on immunisation, but many of us cannot read” (Caregiver 1, Zomba district).

*Distance and logistics in accessing health centres:* Many participants described long distances of travel to the clinics, impacting uptake of vaccination: “Lack of easy access to health centres results in lots of missed immunisation schedules” (Caregivers 1, Zomba District). These sentiments were echoed among all caregivers and community members.

*Disconnect between healthcare system and community gatekeepers/leaders:* Most participants revealed that essential stakeholders (e.g., CL, RL, etc.) were usually not consulted by the EPI and Healthcare System managers. “The EPI does not care about our opinion” (CL, Dowa District). This affected not only attitude, but also the turnout for both RI and adolescent girls for the HPV vaccine.

*Gender role in vaccination decision-making:* Further probing indicated the dominance of husbands in household vaccination decision-making as a factor that prevented turning intentions into behaviour. “Well, even though we know the importance of immunisation, our husbands must still agree before we can carry our children to hospital” (Caregiver 1—Salima District). Another dimension of the gender role viz-a-viz intention and behaviour is that “On major market days, attendance is poor because mothers take husbands’ farm produce to market, so they miss childhood immunisation” (HCW 1; FGD, Dowa District).

### Factors driving hesitancy toward the human papillomavirus vaccine

*Lack of confidence in safety and effectiveness of HPV vaccine:* There were some levels of awareness and even campaigns; however, the communities are not always confident that the HPV vaccine is safe and effective. In many districts, “parents generally, especially fathers, are reluctant to let their eligible daughters receive the HPV vaccine” (HCW 1–2, Lilongwe; Caregivers 1–2, Zomba). “We have heard about the HPV vaccine, but we are not sure about it” (Caregiver 1, Zomba; RL, Lilongwe).

*Complacency:* Caregivers did not believe that cervical cancer was prevalent, because there are almost no cervical cancer screening opportunities outside the main capital. “There is little data to support arguments about high HPV prevalence in our area” (CSO, Salima District). This attitude generated low risk perception of HPV, hence complacent behaviour.

*Lack of awareness of vaccination schedule:* Participants expressed a lack of awareness of the vaccination schedule (dates/timing) as a reason why caregivers missed both routine and HPV vaccinations.

*Knowledge of vaccination versus behaviour:* More than half of the study participants in all four districts acknowledged that there was knowledge of the HPV vaccine; however, this knowledge has not translated into behaviour or uptake. This might be connected to a wide range of issues including beliefs about vaccination in general and specifically about a vaccine that targets young girls (HPV vaccine). “We have not been convinced why the vaccine targets our girls specifically” (Caregivers 2, Salima/Zomba). Therefore, high intentions to vaccinate due to knowledge about the HPV vaccine did not affect uptake behaviour.

*Misconceptions, Rumours, and Conspiracy Theories:* The participants reported a misconception that once their daughters get HPV vaccine, they become infertile. Other caregivers queried why HPV vaccine target only girls. “Ignorance among community members because of rumours about HPV vaccine drives vaccine hesitancy…such as the belief that the HPV vaccine will reduce the libido of girls when they become sexually active and make them become reproductively infertile” (EPI Logistician, Dowa).

*Religious Beliefs:* Participants discussed that there were no widespread traditional or cultural beliefs among Malawian communities that specifically hindered vaccine acceptance. However, there were some specific misconceptions about the vaccine, especially from the Zion and Apostolic faith sects. These groups denied some aspects of modern medicine, including vaccinations, and amplified conspiracy theories about vaccines, such as, vaccines (especially HPV) promote immoral behaviour and leads to infertility among the recipients. “The HPV vaccine promotes promiscuity and exposes young girls to sex and abortion” (RL, Salima/Zomba).

**Fig. 3:**
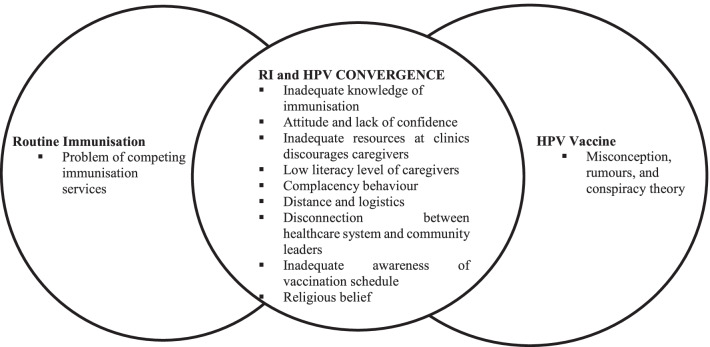
Overview of identified drivers of vaccine hesitancy in Malawi for RI (left), HPV vaccination (right), and both (middle). The data are from both KII and FGD. *RI* Routine Immunisation, *HPV* human papillomavirus

The resulting factors that influenced acceptance or non-acceptance of RI and HPV vaccines are summarized in Fig. 3. Each bubble represents the identified vaccine hesitancy drivers in reference to RI (left), HPV vaccine (right), or both (middle). The figure summarises results from both KII and FGD.

## Discussion

This study identified some key drivers behind vaccine hesitancy in Malawi, focusing on childhood RI and the HPV vaccine. Determinants of vaccine hesitancy included a lack of awareness of the vaccination schedule, lack of trust in the safety and effectiveness of vaccines, complacency, religious beliefs, rumours, and beliefs in conspiracy theories. The study shows that misinformation, rumours around sterility, and reluctance by senior family members or fathers are factors that keep caregivers from vaccination. The absence of accessible evidence or epidemiological data around the disease prevalence supports complacent behaviour among caregivers. However, the study also shows that even correct knowledge and positive attitudes toward vaccination do not reliably turn into actual vaccination behaviour or uptake. Other healthcare activities, such as antenatal care and general out-patient services, are prioritized over vaccination appointments when programs appear to “compete” with each other. Male supremacy in household decision-making can negatively affects vaccination uptake behaviour. But overall, most participants acknowledged that caregivers typically wish for their children to be immunised against vaccine-preventable diseases and agreed that vaccination is a vital topic within households.

Our findings reflected the relatively high national uptake for routine childhood immunisations and indicated that while there is clearly some hesitancy in Malawi, it has not yet translated into widespread declines in childhood vaccination uptake.

The demand for vaccination requires a general perception that vaccines are safe and effective, thereby increasing the feeling of protection from serious illness [32]. This requires that immunisation campaigns be perceived to be of good-quality and have local targets. Given the conservative nature of the setting, fathers or husbands are an important target group that should be given significant priority in educational and advocacy strategies.

Low levels of literacy influence the understanding of public health messaging, and this is most common in caregivers without any formal education who are more likely to miss vaccination clinics [34]. Studies show that insufficient or low literacy is linked to low levels of protective behaviour and can eventually lead to vaccine hesitancy [35]. Our findings here also reflect previous studies across Africa, suggesting that residents with low adult literacy have lower acceptance of vaccination [35]. Vaccination messaging should be target-driven. Written messages directed at communities with low literacy levels should be designed using pictures or symbols that are easy to comprehend. In this regard, an overhaul of the immunisation communication system and educational program of the EPI, which has often focused on urban (high literacy area) compared to rural (low literacy area) settings, must be addressed. Future campaigns should fully consider rural and remote settings in the production planning and dissemination of immunisation knowledge or information, including consideration for local languages or dialects. This study exposed this disparity, and efforts should be geared toward addressing it.

Healthcare-seeking behaviour is driven by numerous factors; for example, our findings show that distance and logistics were not always a primary determinant of vaccination decisions but that the perceived severity of a particular disease also played a part in driving healthcare-seeking behaviour. The decision to forego or miss an immunisation appointment is not determined by circumstances beyond caregivers’ control, but by the convenience of doing it vis-a-vis other commitments and value placed on immunisation. Planning health services is therefore also important. Participants here highlighted that they had to, for example, choose between attending ante-natal or immunisation services. Thus, subsequent national immunisation programs should pay attention to how caregivers can prioritize immunisation while at the same time do not miss other equally important healthcare services. Aligning appointments of competing healthcare services to allow for multiple interventions in one visit, or incentivizing immunisation may help translate caregivers’ intentions into actual behaviour or uptake. Aligning services may also allow outreach services to visit more communities in hard-to-reach areas, shortening travel time and accessing more of the population.

Communication with the local “gatekeepers” (for example, CL and RL) is critical when building and strengthening co-operation. It boosts local confidence in healthcare services including vaccination. Also, integrating a Short Message Service (SMS) reminder system will go a long way in addressing the vaccination schedule problems identified among caregivers (i.e., for those who have mobile devices) [36, 37].

Vaccine hesitancy, particularly for HPV, is prevalent among some Malawian communities regardless of their cultural or religious affiliations. In Rumphi and Zomba districts, some parents declined consent for their daughters to receive the vaccine, because they believe that immunisation is dangerous [10]. Some misconceptions about the HPV vaccine can be corrected by RL, including the Zion and Apostolic Christian faith sects who continue to doubt the safety of vaccines. In the case of the polio eradication program in countries like Afghanistan, Pakistan, and Nigeria, a small number of local Muslim leaders have sometimes convinced their followers that “it is an American ploy to sterilize the Muslim communities” [38]. It is important for proactive health promotion campaigns to understand and appropriately counter these sentiments. Therefore, efforts of intervention in Malawi must be directed at opinion leaders and gatekeepers, especially CL and RL, who wield strong influence to change the narrative.

The uniqueness of this study and its contribution to knowledge are centred around understanding nuances of vaccination acceptance or vaccine demand insights and the underlining drivers in resource-low settings such as SSA e.g., Malawi, where there had been dearth of evidence-based findings for policy actions. The literature review had significant exposed this wide vacuum, where evidence-based finding on this subject have been largely situated in HIC. Hence, this study will help the national EPI and international public health intervention organizations in SSA to increase knowledge on enablers of vaccine hesitancy or burden of vaccine-preventable-diseases in the region. By using three multilayer levels model advanced by WHO (e.g., vaccines/vaccination-specific-issues, individual and group influence and contextual), this study will help public health intervention agencies and national/sub-national policy actions to be target-specific, measure-defined and avoid using approaches meant for HIC in low-resource settings such as SSA.

While the study provided behavioural insights regarding drivers of vaccination decision-making in Malawi, there is need for further quantitative analysis on the prevalence of the determinants, as well as their causal relations to vaccine uptake.

The main limitation experienced during this study was the problem of the COVID-19 pandemic, which resulted in unanticipated travel restrictions. This forced the study to substitute two of the originally planned districts (Nsanje and Rumphi) for convenient ones (Salima and Lilongwe) to enable swift and easy access to the data collection sites due to travel distance. Second, interviews at the central level (Lilongwe) took longer than expected to complete, because some participants were COVID-19 essential-services personnel and found it difficult to make time for interviews. Third, participation among caregivers was low compared to other stakeholders. Lastly, additional studies are necessary to understand the perspectives of the ultimate decision-makers of vaccination, especially fathers. Overall, the limitations were minimal, partly because the infection rate in Malawi during the data collection in March/April 2020 was not significant and COVID-19-related restrictions were not yet fully active in most districts. Also, since the study target was a mixture of opinion, the caregiver’s number was sufficient.

## Conclusion

The evidence presented here, and the lessons learnt from the roll-out of new vaccines such as against HPV can provide a starting point for tailored public health messaging that are specific to the Malawi population. This has implications both for current levels of vaccine acceptance and the introduction of any new vaccine, such as the vaccines against Malaria, HIV/AIDS or COVID-19. Although more studies are still required in these areas.

The study shows that a network of factors determines vaccine hesitancy for RI and HPV, and some of them are interrelated with one another. Strategies developed to address vaccine hesitancy must be multicomponent and wide-ranging. Invariably, the factors that lower the demand for childhood RI are also key to low demand for the HPV vaccine and vice versa. For the introduction of the new COVID-19 vaccines, the following will be especially important: considering the literacy level of the population and allowing the communication campaigns to be sensitive to local settings; ensuring that messaging on safety and vaccine effectiveness are driven by gatekeepers and RL, especially from the most sceptical Christian sects; dealing with low risk perception and conspiracy theories inspired by rumours and misinformation by using local celebrities or credible community gatekeepers. A proactive and coordinated approach to health promotion will be vital in ensuring high levels of acceptance and increased uptake.

## Data Availability

The datasets used and/or analysed during the current study are available from the corresponding author on reasonable request.

## Abbreviations

AMP Afrique: Agence de médecine preventive afrique
CEREB: Center for empirical research in economics and behavioural sciences
CL: Community leaders
CSO: Civil society organisation
DPT3: Third dose of diphtheria, tetanus, and pertussis vaccine
EPI: Expanded program on immunisation
FGD: Focus group discussion
GAVI: Global alliance for vaccine and immunisation
GHP3: Global health population, poverty, and policy
HCW: Healthcare workers
HPV: Human papilloma virus
IARC: International agency for research on cancer
KII: Key informant interview
NHSRC: National health science research committee
NITAG: National immunization technical advisory group
RI: Routine immunisation
RL: Religious leaders
SDG: Sustainable development goals
SSA: Sub-Saharan Africa
UHC: Universal health coverage
UNICEF: United Nations Children Emergency Fund
VPD: Vaccine preventive disease
WHO: World Health Organisation
WUENIC: WHO UNICEF immunisation coverage estimates
